# The potential for plant growth-promoting bacteria to impact crop productivity in future agricultural systems is linked to understanding the principles of microbial ecology

**DOI:** 10.3389/fmicb.2023.1141862

**Published:** 2023-05-19

**Authors:** Salme Timmusk, Taavi Pall, Shmuel Raz, Anastasiia Fetsiukh, Eviatar Nevo

**Affiliations:** ^1^Department of Forest Mycology and Pathology, Uppsala BioCenter, Swedish University of Agricultural Sciences (SLU), Uppsala, Sweden; ^2^Estonian Health Care Board Department of Gene Technology, Tallinn, Estonia; ^3^Department of Information Systems, University of Haifa, Haifa, Israel; ^4^Institute of Evolution, University of Haifa, Haifa, Israel

**Keywords:** symbiotic extended phenotypes, native, harsh, and wild agricultural systems, hologenome, horizontal DNA transfer, DNA methylation, core microbiome

## Abstract

Global climate change poses challenges to land use worldwide, and we need to reconsider agricultural practices. While it is generally accepted that biodiversity can be used as a biomarker for healthy agroecosystems, we must specify what specifically composes a healthy microbiome. Therefore, understanding how holobionts function in native, harsh, and wild habitats and how rhizobacteria mediate plant and ecosystem biodiversity in the systems enables us to identify key factors for plant fitness. A systems approach to engineering microbial communities by connecting host phenotype adaptive traits would help us understand the increased fitness of holobionts supported by genetic diversity. Identification of genetic loci controlling the interaction of beneficial microbiomes will allow the integration of genomic design into crop breeding programs. Bacteria beneficial to plants have traditionally been conceived as “promoting and regulating plant growth”. The future perspective for agroecosystems should be that microbiomes, via multiple cascades, define plant phenotypes and provide genetic variability for agroecosystems.

## 1. Introduction

By the end of the century, crop production will need to increase by 50% to meet the anticipated food demand and encounter the challenges caused by climate change (Morales Moreira et al., 2022; U.S. Department of Agriculture, 2022). The genetic diversity of an ecosystem has become a biomarker for its health as it optimizes microbial functions and leads to strong ecosystem complementarity (Langenheder et al., 2010; Berg et al., 2017; Perez-Jaramillo et al., 2018). Climate change impacts soil and its biodiversity, and this affects the health of the ecosystem, which in turn impacts food production (Morales Moreira et al., 2022; U.S. Department of Agriculture, 2022). During the green revolution from 1950 to 1984, agricultural production increased substantially to meet the demand for food at the time, but the strategies used created simplified agroecosystems that replaced biological functions originally present in native communities (Bommarco et al., 2013). Indeed, global agricultural production was significantly increased by adopting large-scale monocultures, applying massive amounts of synthetic fertilizers and pesticides, and restricting gene pools through selective breeding (Averill et al., 2022). The downside of this enormous program was the creation of agroecosystems with low genetic diversity more susceptible to extreme climate effects (Bommarco et al., 2013). It is generally accepted now that land use intensification is the most important global change (Wang et al., 2022). How should we ensure the enhanced food production needed to feed the increased population at the end of the century? It can be demonstrated by experimental manipulation and meta-analysis combining multiple scientific studies that microbiome diversity and network complexity in native ecosystems enhance multiple functions in the systems, and this generates a more secure production (Wittebolle et al., 2009; Wagg et al., 2014; Morrien et al., 2017). Owing to the recent advances of OMIC technologies, the omnipresence of microbial symbioses with plants has been repeatedly confirmed, showing that many host phenotypes are in fact symbiotically extended phenotypes (Lynch and Hsiao, 2019; Batstone et al., 2020, 2022; Batstone, 2022). These symbiotic extended natural populations in native environments studied by genome-wide association mapping reveal the genes involved in the symbiosis (Batstone et al., 2020, 2022; Batstone, 2022). The mutualistic cooperation between plant hosts and microbes acts to sustain genetic diversity, partially explaining why variations in mutualism traits are stable in nature (Batstone et al., 2022).

Here, we argue that understanding and integrating co-evolutionary principles and genetic and molecular mechanisms of native ecosystems will help us design and holistically predict the consequences of microbial symbiosis in modern agroecosystems. Adoption of natural measures will reduce the requirement for external inputs, and instead, we can rely on the biological functions and biodiversity originally provided by native communities.

## 2. Microorganisms are abundant in soils and influence plant biotic and abiotic conditions

Microorganisms represent the largest fraction of global biomass (15% of the total living biomass) as well as most of the global diversity (Bar-On et al., 2018; Averill et al., 2022). Microorganisms are abundant in soils, with up to 10^9^ cells per gram comprising up to 10^6^ taxa. Microbial life determines the physical, chemical, and biological characteristics of soil ecosystems (Bar-On et al., 2018; Averill et al., 2022). Bacteria are usually the dominant microorganisms (90%) in soil, contributing more biomass than protists and archaea (Timmusk et al., 2017). Thus, soil is a major source of microorganisms in terrestrial ecosystems. The definition of rhizosphere was introduced by Hiltner in 1904 and is the area around a plant root that is inhabited by a unique population of microorganisms that influences the plant root (Hiltner, 1904). The commercial development of inoculants began more than 100 years ago (Bashan, 1998). The plant growth-promoting bacteria (PGPB) are the best-studied group within the plant microbiome. It has long been known that some bacteria influence plant biotic fitness (Wiley, 1902). Rhizobacteria can promote plant growth either directly or indirectly. Initially, it was believed that the aid to the plant from PGPB is limited to acquiring essential nutrition, such as that from nitrogen, phosphorus, and other essential minerals, or reducing the actions of pathogens that inhibit plant growth (Hamazaki et al., 1950; Moores et al., 1984; Moore, 1988). A change in paradigm occurred in 1999 when it was discovered that, in addition to fighting biotic stresses, PGPB can influence plant abiotic stress conditions by enhancing desiccation tolerance (Timmusk and Wagner, 1999), a few years later, it was reported that rhizosphere bacteria can alleviate salt stress (Mayak et al., 2004). Bacterially induced gene expression patterns suggested a connection between plant abiotic and biotic stress regulation (Timmusk and Wagner, 1999). These discoveries opened a new era of research focusing on rhizosphere bacteria that helped plants to settle in unfavorable environments. The large number of publications on PGPB demonstrates the growing interest in supporting their use in agriculture (for reviews, see Glick, 2012; Timmusk et al., 2017; De-Bashan et al., 2020; Adedayo et al., 2022; Gamalero and Glick, 2022). Comprehensive evaluations of the potential of rhizobacteria to restore the environment via phytoremediation, phyto-transformation, and bio-augmentation, all leading to a healthier environment, have also been published (de-Bashan et al., 2012; Timmusk et al., 2021; McCorquodale-Bauer et al., 2023). Owing to the reduced cost in recent years of multi/OMICS technologies, we have realized that soil is a highly heterogeneous growth medium and microbial populations fluctuate in space and time owing to the variable environmental conditions (Langenheder et al., 2010; Prosser, 2013; Timmusk et al., 2018; Ray et al., 2020).

The problem with applying PGPB products has been limited persistence under field conditions (Timmusk et al., 2017; Kaminsky et al., 2019). The products in the natural environment often do not provide the same benefits as they do under controlled conditions (Timmusk et al., 2017, 2018; Dini-Andreote and Raaijmakers, 2018; Oyserman et al., 2018; Kaminsky et al., 2019; Ray et al., 2020; Trivedi et al., 2020, 2021; Timmusk and de-Bashan, 2022). The reason is that PGPB strains are being outcompeted by native communities, or their colonization and active principles are reduced to ineffective levels (Bar-On et al., 2018; Averill et al., 2022). This is the evidence that crop plant microbiome association selections and evaluations have been primarily based on taxonomic/qualitative criteria, and lack microbiome-associated plant phenotypes qualitative trait-based quantitative analyses (Oyserman et al., 2018). Several approaches such as novel formulation strategies, the development of endophytes colonizing plants and seeds, repeated inoculations, and host-mediated engineering have been used to increase PGPB persistence in natural systems (Del Barrio-Duque et al., 2019; Kaminsky et al., 2019; Sessitsch et al., 2019; French et al., 2021). Here, we explore a “back to the roots” approach, studying the microbial community and plant complementarity traits from indigenous communities (Dini-Andreote and Raaijmakers, 2018; Oyserman et al., 2018; Siegel-Hertz et al., 2018; Kaminsky et al., 2019; French et al., 2021).

## 3. A systematic approach for the identification of the microbiomes of ecologically and economically important plant species

**Figure 1:**
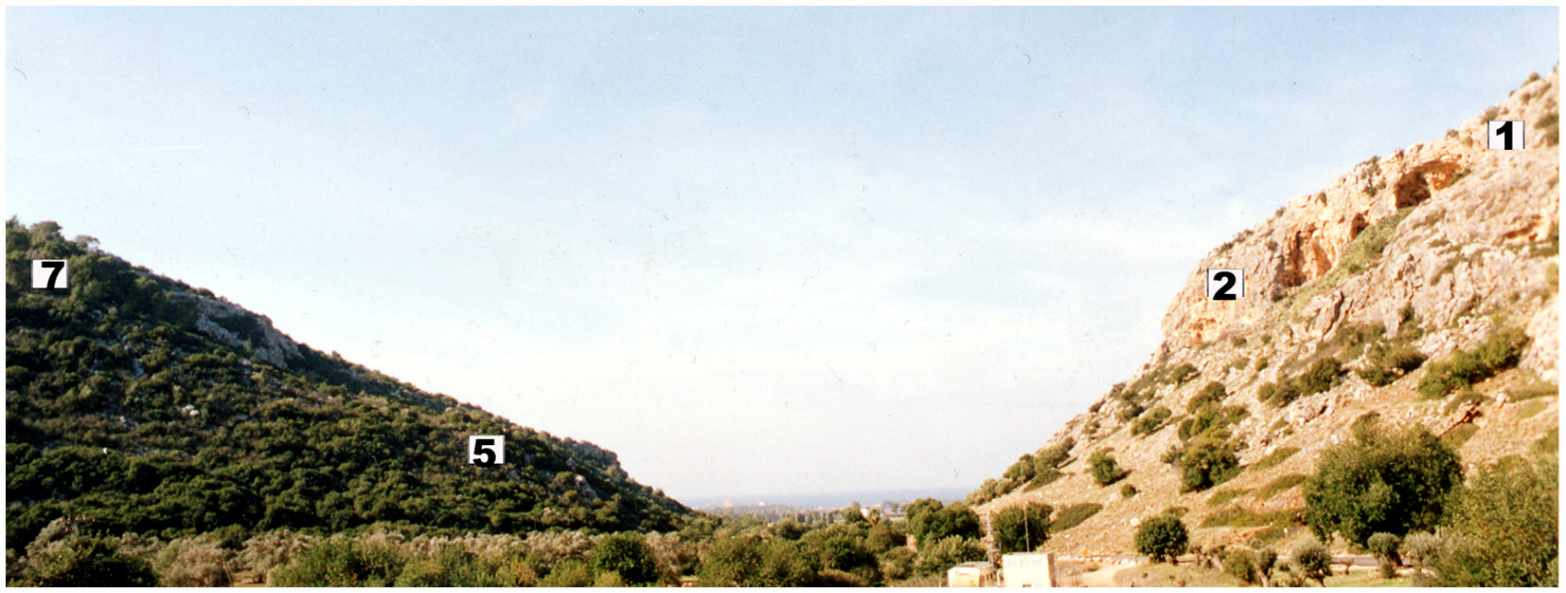
Cross-section of the “Evolution Canyon” indicating the collection sites on south-facing slopes (SFSs) 1 and 2 and north-facing slopes (NFSs) 5 and 7. Figure taken by Nevo (2012).

The beneficial, neutral, and pathogenic microorganisms are the compartments of plant microbiota. However, although individual members of plant-associated microbial communities can possess certain beneficial traits, the manifestation of a trait in the community is an emergent property that cannot be predicted by the individual members. We have learned that mixed PGPB consortia of compatible microorganisms mutually optimize functions that lead to stronger ecosystem complementarity (Timmusk et al., 2018; Ray et al., 2020). Over the decades, PGPB mechanisms of growth promotion have been explored, we can regard the benefits as largely direct or largely indirect. The direct benefits include the production of phytohormones, the production, transformation, and translocation of critical nutrients, and the alleviation of environmental stresses. PGPB can promote plant growth indirectly through the enhancement of a plant's resistance responses, competition for nutrients and niches, and protection from plant pathogens through competition and antibiosis (Figure 1) (Timmusk, 2003; Glick, 2012). Since plant traits are usually coregulated by the plant-associated microbiome, there is an emerging theory that the plant microbiome generates new phenotypes with increased fitness under distinct environmental conditions. In this context, the interactions between plants and their associated microbiome should not be considered inherently either beneficial or deleterious (Ravanbakhsh et al., 2019). The paradigm is the basis of the rationale for designing synthetic communities of microorganisms with wide-ranging, consistent, and long-lasting plant growth-promoting traits. While linking traits to ecosystem processes referring to genotype interactions in complex communities have been discussed for a long time, recently, the principles behind the systematic screening of the genetic potential of ecosystems, including the design of microbial consortia, have been comprehensively described (Oyserman et al., 2018). Ecosystems are seen as reservoirs of genetic potential that may be mined for identifying microbiome-associated phenotypes (MAPs). MAPs are systematically screened and quantified to identify instances (e.g., plant, microbe, and environmental combinations) in which MAPs provide the largest fitness advantage (Oyserman et al., 2018).

Insights into the complex interactions of traditional, wild, and harsh ecosystems will help improve our understanding of the evolutionary and ecological diversification that controls and improves plant fitness (Filho et al., 2023).

## 4. Traditional agricultural practices, and wild and harsh habitats

It is generally known that the use of intercropping, crop rotations, and manure and compost treatments, which are essential elements of ancient and traditional agricultural practices, have significant benefits for microbial biodiversity. The positive effect of the practices of crop production is often related to biodiversity (Kim et al., 2020; French et al., 2021). Alteration of the microbiome structure, the sustainable growth of beneficial bacteria, and the growth reduction of phytopathogens are the result of methods used in traditional agricultural practices (Kim et al., 2020; French et al., 2021). The research focused on the methods has suggested various mechanisms behind these effects (Bonanomi et al., 2018; Wang et al., 2020). For example, the promotion of pathogen growth suppression can occur when there is a shift in pH (Bonanomi et al., 2018; Wang et al., 2020). Chitin and keratin are molecules that when present, may act as the enrichment of microorganisms that suppress the degradation of these compounds, at the same time suppressing fungal pathogens of which the cell walls are composed of chitin and keratin (Bonanomi et al., 2018; Wang et al., 2020; Andreo-Jimenez et al., 2021). An increase in the Pseudomonads biocontrol strains is the result of practices that increase soil amino acids and long-chain fatty acid contents (Wen et al., 2021).

In wild environments, microbes adopt diverse mechanisms that coordinate community activity and enable complex multi-cellular processes (Langenheder et al., 2010; Gilbert, 2016; Timmusk et al., 2017; Perez-Jaramillo et al., 2018; Kaminsky et al., 2019; Blanchet et al., 2020; Gilbert and Hadfield, 2022; Timmusk and de-Bashan, 2022). It is suggested that plants have co-evolved with microbes for millions of years and that this may have enabled plants to colonize land (Pirozynski and Malloch, 1975). Transitions of plants from their native habitat to agricultural soil lead to substantial changes in the microbiomes (Perez-Jaramillo et al., 2018; Raaijmakers and Kiers, 2022). Wild relatives of crop plants in associations with microorganisms, resembling the need of humans for a microbiome, can respond to changing environmental conditions and occupy extreme habitats (Timmusk et al., 2017; Perez-Jaramillo et al., 2018; Timmusk and de-Bashan, 2022). Plant genotypes and the microbiome composition of wild relatives have evolved over long periods of time, together determining the diversity and stress tolerance in the centers of crop plant origin (Timmusk et al., 2011, 2014; Bulgarelli et al., 2015; Perez-Jaramillo et al., 2018; Timmusk and de-Bashan, 2022).

An extreme or harsh environment is a habitat characterized by harsh environmental conditions beyond the optimal range for the development of humans, e.g., pH 2 or 11, −20 or 113°C, saturating salt or technogenic concentrations, high radiation, or 200 bars of pressure. Microbiomes of harsh habitats (extremophiles) are known to function in many ways to improve plants' capacity to counteract stress situations.

Here we propose that traditional farming systems, wild and harsh habitats genetic diversity could be studied as reservoir for mining microbial associations for plants' fitness under distinct environmental conditions.

One such wild and harsh center is the well-described ecological laboratory called Evolution Canyon (EC) found in northern Israel (Sikorski and Nevo, 2005; Timmusk et al., 2011; Nevo, 2012) (Figure 1). The “African” or south-facing slopes (AS or SFS) in canyons north of the equator receive higher solar radiation than on the adjacent “European” or north facing slopes (ES or NFS). The difference in solar radiation causes higher maximal and average temperatures and evapotranspiration on the more stressful “African” slope. This results in dramatically diverging physical and biotic interslopes, probably originating several million years ago, after mountain uplifts. These canyons are remarkable natural evolutionary laboratories. While microclimate remains the major interslope divergent factor, geology, soils, and topography are similar on opposite slopes (50–100 m apart at the bottom). Thus far, to unravel the link between environmental stress and adapting genome evolution, the intraspecific interslope divergence has been compared in 2,500 species across various life forms from prokaryotes to eukaryotic lower and higher plants, fungi, and animals. The special features of the ecology facilitate drawing up models of biodiversity and genome evolution from which follow testable predictions (Sikorski and Nevo, 2005; Timmusk et al., 2011; Nevo, 2012).

## 5. Mechanisms that generate biodiversity in the rhizosphere

**Figure 2:**
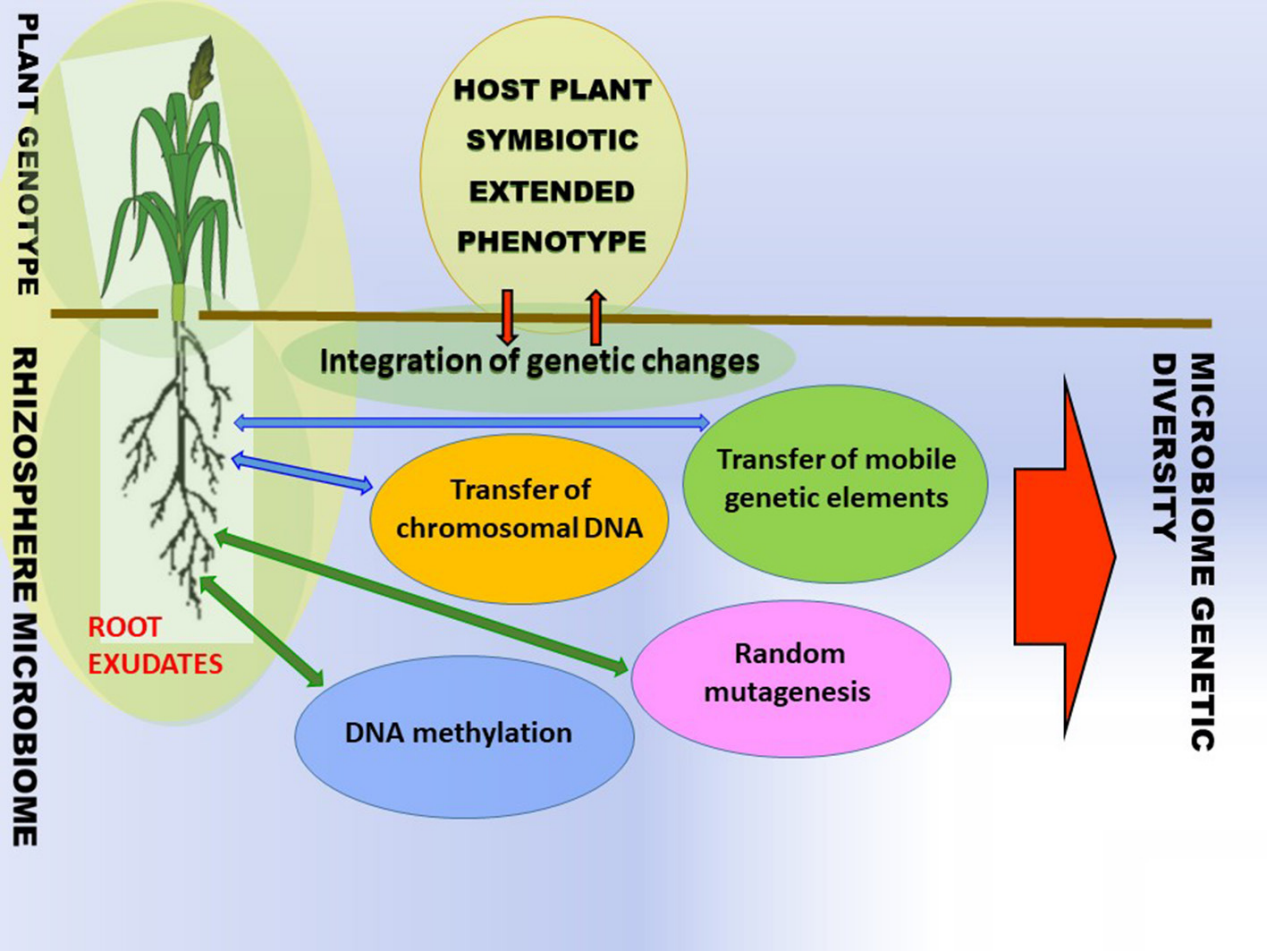
Ecosystems are reservoirs of genetic variability. The phenotypic variance of plant traits is induced (i) via nested interaction, i.e., with microbe chromosomal DNA, mobile genetic elements (ii) directly with mobile genetic elements or chromosomal DNA in soil or (iii) host plant DNA methylation (iv) bacterial random mutagenesis. Microbial variance is induced via the transfer of mobile genetic elements, chromosomal DNA, and random mutagenesis.^1^ ^1^Environmental variance components are excluded for simplicity. Adapted by Batstone (2022) and Timmusk et al. (2017).

While it is generally known that plants are colonized and influenced by a plethora of microbes, the sources and mechanisms of intra-species variation of bacterial and host plant traits are not well-understood. Bacteria are susceptible to modifications that lead to the emergence of new genetic variances. The modifications can either be short-term adaptations or long-term evolution. The modifications can occur in the form of random mutagenesis, horizontal transfer of chromosome DNA, and transfer of mobile genetic elements (MGEs) (Figure 2). The suggested common name of the event is horizontal gene or horizontal gene (DNA) transfer (HGT or HDT) (De La Cruiz and Davies, 2000; Shapiro, 2021). The genetic changes in rhizobacteria and plants can be acquired through various mechanisms including, but not limited to, HGT (Batstone, 2022) and DNA methylation and random mutagenesis (Figure 2) (Wall et al., 2015; Gilbert and Hadfield, 2022; Shapiro, 2022).

### 5.1. Horizontal gene transfer (HGT)

Horizontal gene transfer, the transmission of DNA between different genomes of different species, occurs by three genetic mechanisms: transformation (bacteria take up DNA from their environment), conjugation (bacteria transfer genes directly to another cell), and transduction (bacteriophages move genes from one cell to another). HGT is best known in prokaryotes, causing major challenges for bacterial taxonomy (Prosser et al., 2007). The bacteria can transfer a small part of one genome to replace a homologous region in another genome, without disrupting the integrity of the species. Furthermore, HGT can also lead to gene transfer with no counterpart in the recipient. The genetic information can be maintained on a plasmid or integrated by non-homologous recombination such as plasmids (extrachromosomal genetic material), transposons (“jumping genes”), and bacteria-infecting viruses (bacteriophages). The bacterial genome, as a result of gene transfer, can consist of two distinct parts, called the core genome and the accessory genome. The housekeeping genome consists of genes that may be regarded as essential for the species to exist (Prosser et al., 2007). In contrast, the accessory genome contains genes encoding special ecological adaptations that are easily gained or lost. Strains belonging to the same species, as defined by 16S ribosomal RNA (rRNA) sequences, can differ according to whether they possess hundreds of accessory genes that potentially adapt them to different ecological situations (Prosser et al., 2007). Surveys of rRNA gene sequences demonstrate the vast diversity of bacterial communities, but if the accessory genome confers many of the important ecological adaptations, then the true capacity for ecological diversity exists in the “rich brew of catabolic plasmids, resistance transposons and genetic islands” (Prosser et al., 2007), which are almost certainly gained from bacterial biofilms in harsh habitats (Timmusk et al., 2011, 2014). As a result, taxonomically unrelated bacteria can share an accessory genome, advantageous in a particular environment while being absent from the “same” bacterial species growing elsewhere (De La Cruiz and Davies, 2000; Timmusk et al., 2011).

How do plants adapt to changing environments? It is generally known it happens via changes in plant gene expression and genomic rearrangements for new functions e.g., gene duplications. The rearrangements also happen in the form of the acquisition of exogenous genes for new functions via HGT. It has been proposed that mobile elements produce major evolutionary leaps in eukaryotes, similar to the way how bacterial mobile elements produce speciation via the same HGT mechanisms (De La Cruiz and Davies, 2000). Massive endosymbiotic bacteria in the nucleus led to the origin of eukaryotes (De La Cruiz and Davies, 2000). HGT transfers may cause major adaptive changes and involve DNA segments encoding whole proteins, or encode only a few individual domains, and have been documented across virtually all taxonomic units (Shapiro, 2022). Identification of HGT events can be performed by comparing the single-nucleotide polymorphism patterns of pangenomes, or coding sequence compositions, gene phylogeny, and genome compositions. The comparative analysis shows that even though HGT-mediated microevolution takes place in all environments, HGT is considered the evolutionarily important mechanism under situations of selection pressure in harsh environments or population interactions over long periods of time (Shapiro, 2021, 2022). Thus, rhizobacteria of the wild relatives at the centers of origin can be seen as mediators of plant and ecosystem genetic diversity (Bashan, 1998; Timmusk et al., 2011, 2014; Perez-Jaramillo et al., 2018; Timmusk and de-Bashan, 2022).

### 5.2. DNA methylation

Enhanced DNA methylation is to be regarded as an evolutionary driver priming for enhanced defense response against abiotic stresses (Chen et al., 2022; Tomczyk et al., 2022). Bacterially mediated plant DNA methylation can affect gene expression and transcriptional repression, as well as DNA replication (Timmusk and de-Bashan, 2022). Several studies of microbe–plant interactions highlight modifications of DNA methylation in roots after bacterial application and rhizobacterially induced DNA methylation in roots can promote plant growth and ecosystem diversity. Importantly, these epigenetic modifications functioned even after the inoculum was removed from the microbiome (Chen et al., 2022). For example, the upregulation of DREB2A and P5CS in PGPB-primed stressed plants confirms that PGPB priming increased drought tolerance via osmoprotection (Lephatsi et al., 2022). Measurements of the global DNA methylation confirmed the priming phenomenon as involving epigenesis. Global DNA methylation levels in PGPB-primed plants increased under both mild and severe drought stress conditions (3.7- and 6.4- fold; 9.2 and 21.5%, respectively) and this indicated restored genomic integrity resulting in drought stress tolerance (Lephatsi et al., 2022).

## 6. Engineering contemporary crop plant microbiomes

The aforementioned HGT and DNA methylation, along with random mutagenesis, diversify ecosystems and lead to the generation of novel bacterial and plant genotypes which on a large scale contribute to ecosystem biodiversity (Figure 2). To adapt the plant variance formula (Batstone, 2022), phenotypic variance of plant traits = genetic variance in the host plant + genetic variance in the microbe + genetic variance in the HGT+ genetic variance by host plant DNA methylation A general biodiversity principle is that different organisms enhance productivity and the functioning of ecosystems by utilizing different resource pools due to the different life strategies. Diverse symbionts are mediators of plant diversity as they stimulate agroecosystem functioning by supplying different services, e.g., limiting nutritional elements and osmolytes by supporting different plant species (Figure 2).

The plants with symbiotic extended phenotypes in traditional agricultural practices and harsh and wild habitats can act as holobionts composed of numerous genetic lineage interactions with other organisms, and these are crucial for the development and maintenance under stress situations (Gilbert, 2016; Timmusk et al., 2017; Gilbert and Hadfield, 2022; Timmusk and de-Bashan, 2022). The critical question is: what are the vital mechanisms that microorganisms use for interaction with the host plants? Plant microbial communities are assembled according to the plant genotype-based root exudation and surrounding microbiome composition (Figure 2). Can modern plant species be less susceptible to environmental stresses when associated with an appropriate microbiome from wild relatives, harsh habitats, or traditional agricultural systems? It certainly is not an easy question to answer. In the process of crop plant domestication, crop plants have acquired new traits (e.g., taste, larger seeds) which eventually secured better food supply and reduced susceptibility to pathogens. At the same time, their microbiome composition changed, and restricted gene pools caused by crop plant breeding traditions resulted in the selection of domesticated genotypes, which might have eroded plant traits with functions interacting with the root microbiome. Therefore, transferring complex microbiomes may be just the initial start-up and may need to be re-evaluated using different breeding lines, eventually finding strategies for synthetic microbiome associations with plant genotypes with a positive response (Raaijmakers and Kiers, 2022). First, the selection of plant fitness and productivity associated microbiome should be performed and the key microbes identified based on integration network structural and functional models (Oyserman et al., 2018). Individual microbial strains are then cultured and characterized isolates are screened using high-throughput platforms and further validated for plant growth promotion in phytotrons or standardized fabricated microcosms. The most promising consortia are applied to fields using high-throughput monitoring systems (Dini-Andreote and Raaijmakers, 2018; Oyserman et al., 2018).

## 7. Challenges with technologies

The confluence of technological advances makes it feasible to uncover the mysteries of plant–microbial interactions in natural systems. The purpose of this review is not to comprehensively describe all challenges that accompany OMICS and other technology applications, as reviews on technical and bioinformatic limitations are already published (Wooley et al., 2010; Morales and Holben, 2011; Carvalhais et al., 2012; Prakash and Taylor, 2012; Temperton and Giovannoni, 2012; Prosser, 2015). Here, we attempt to emphasize some challenges that occur in the microbiomes of harsh environments based on PGPB product development. First, problems associated with cell lysis and nucleic acid extraction are common but much more so in harsh environment soils (Sessitsch et al., 2019; Timmusk and de-Bashan, 2022). Therefore, it has to be considered that when isolating nucleotides from the soils, complete coverage may not be obtained (Prosser, 2020).

It is generally known that plants select microbiomes based on functional traits rather than taxonomy. Hence, the traits provided by microbiomes are more informative than taxonomic information. The current microbial taxonomic approach is high-throughput 16S rRNA sequencing. However, as discussed in relation to HGT, considering the fluid nature of prokaryotes, the taxonomy of core microbiomes remains challenging and may not reflect the diversity of the rhizosphere beneficial to plants. HGT at all phylogenetic levels prevents consistent taxonomic definition and it is important to understand these limitations. Therefore, it has been suggested to focus on the bacterial consortia with a similar function, i.e., phylotypes that specify the core microbiome as a temporal, ecological, and functional core (Bonanomi et al., 2018; French et al., 2021).

The recent development and application of PGPB products have shown that taxonomically highly abundant representatives are chosen for inoculation to promote plant growth and metagenomics data and are usually presented as a relative abundance of phylogenetic or functional genes (Armanhi et al., 2016, 2017; Del Barrio-Duque et al., 2019). Abundance-driven measures mean that the different limiting values include taxa or functional genes with various levels of associations with the experimental/environmental criterion. While different ecological roles and methods that combine abundance and occurrence have been suggested, it should be considered that sometimes the abundant associations could simply reflect repeated acquisitions of microbes from the environment by the host rather than functional key genera. Within the abundant microbiota, less abundant organisms can influence the interactions between the host and other microbiota and have a regulatory effect on the network of interactions (Blanchet et al., 2020).

Different approaches have been proposed to determine taxa with potential key ecological functions in agro-ecological systems, and network analysis has been applied to statistically determine the influential taxa (Armanhi et al., 2016, 2017; Banerjee et al., 2018). Correlation networks are produced by correlating abundance patterns from gene-targeted or metagenomic sequencing data [26]. Considering the complexity of most microbial habitats, there are serious limitations to direct evaluation and validation of the influential taxa (Rivett and Bell, 2018; Blanchet et al., 2020; Guseva et al., 2022). Hence, questions such as how strong the interaction signals are, what are the relevant co-variants, and how important are the detection errors should be asked to discover if the putative core is a stable component of the host (Blanchet et al., 2020). Furthermore, it should be considered that gene copy number varies between different taxa, and the number per cell may also vary within a single organism under different growth conditions (Chai et al., 2011). Therefore, caution is required when studying gene-centric links between relative abundance and phylotypes and their functional activity. Considering the limitations, it has been suggested that gene-centric metagenomics should be replaced with genome-centric metagenomics, e.g., correlations between phylogenetic (16S rRNA) and functional genes could be studied, as the combination of genome-centric metagenomics and meta transcriptomics can indicate the metabolic bases for the adaptation of taxa (Prosser, 2015). These changes and correlations, in turn, may indicate important ecological adaptation mechanisms and present meaningful targets for PGPB research.

Metagenome- and genome-wide association studies (MWAS/GWAS) are traditionally used to predict functional traits enriched in the presence of microbiome association communities. While the studies have identified key drivers for the assembly of plant-associated microbiota (Trivedi et al., 2020) they indicate that large proportions of the variation in community assembly and the effects of microbiomes is still not explained (Trivedi et al., 2020). Large-scale MWAS/GWAS approach considering indigenous, traditional and harsh 387 environments genetic diversity should be instrumental to elucidate these gaps.

Techniques are available to characterize the vast diversity in microbial communities, but the challenge is to identify key questions and address them with sound conceptual approaches and appropriate techniques, including an understanding of the limitations.

## 8. Concluding remarks

Microbiomes beneficial to plants operate through diverse mechanisms that depend on a complex network of evolutionary and ecological factors. Therefore, modern agricultural practices will benefit from incorporating ecological and evolutionary principles of native, wild, and harsh environments. This approach would help understand the mechanisms that influence plant microbiome interactions in ecosystems and allow the development of new tools to mitigate biodiversity loss and ensure the resilience and sustainability of agroecosystems.

The primary objective of this analysis is not to view the discussion topics in an all-inclusive manner, but rather to encourage thought and consideration for novel perspectives when exploring plant–microbe interaction studies.

## Author contributions

ST and TP: conceived, designed, and wrote the manuscript. EN, SR, and AF: discussed the manuscript. All authors contributed to the article and approved the submitted version.
